# The Impact of Step Recommendations on Body Composition and Physical Activity Patterns in College Freshman Women: A Randomized Trial

**DOI:** 10.1155/2019/4036825

**Published:** 2019-12-01

**Authors:** Bruce W. Bailey, Ciera L. Bartholomew, Caleb Summerhays, Landon Deru, Sharla Compton, Larry A Tucker, James D. LeCheminant, Joseph Hicks

**Affiliations:** ^1^Department of Exercise Sciences, Brigham Young University, Provo, UT, USA; ^2^Department of Nutrition, Dietetics & Food Science, Brigham Young University, Provo, UT, USA

## Abstract

**Purpose:**

Transitioning from high school to college generally results in reduced physical activity and weight gain at a rate that is higher than the general population. The purpose of this study was to examine the effects of three progressively higher step recommendations over 24 weeks on changes in body weight and body composition.

**Methods:**

Ninety-two freshmen college women wore a multifunction pedometer for 24 weeks after being randomly assigned to a daily step level: 10,000, 12,500, or 15,000. Pedometer data were downloaded every two weeks and participants were counseled on meeting their step recommendation. Body weight and body composition were assessed at baseline and 24 weeks. Body composition was assessed by dual X-ray absorptiometry.

**Results:**

On average, women took 10,786 ± 1501, 12,650 ± 2001, and 13,762 ± 2098 steps per day for the 10,000-, 12,500-, and 15,000-step groups, respectively (*F* = 15.48, *P* < 0.0001). Participants gained 1.4 ± 2.6, 1.8 ± 2.1, and 1.4 ± 2.1 kg for the 10,000-, 12,500-, and 15,000-step groups, respectively (*F* = 37.74, *P* < 0.0001). Weight gain was not significantly different between groups (*F* = 0.18, *P*=0.8385). There was also no difference in fat weight gain (*F* = 0.41, *P*=0.7954).

**Discussion:**

A step recommendation beyond 10,000 does not prevent weight or fat gain over the first year of college. Future research should focus on either intensity of physical activity or the addition of dietary interventions to prevent weight gain during the first year of college.

## 1. Introduction

College students represent a high-risk population for weight gain and obesity (1–3). This is partly due to the fact that many college students are in a critical transitional life stage and are establishing patterns that may persist throughout adulthood [1]. Studies of college students indicate that a 1–4 kg weight gain is common during their first 2 years of college with a recent prospective study suggesting some students gain as much as 20.8 kg over the course of four years of college [2, 3].

Though not always a straightforward relationship, physical activity has been shown to be significantly associated with weight and body fat in women [4–7]. Reduced physical activity has a negative impact on energy balance and thus may lead to weight gain if dietary compensation does not take place. Physical activity can prevent weight gain by increasing energy expenditure, thereby adjusting for small positive energy imbalances [8]. Physical activity tends to decline in the transition from high school to college, [9] and this decline could partially explain the observed weight gain during the first years of college.

Using a pedometer to count steps is an easy method to track and promote physical activity [10]. There is a growing body of research demonstrating the ability of pedometers to promote activity, including one investigation which showed that participants increased the number of steps taken by 26.9% [11, 12]. These same studies also demonstrated that pedometers can be used to decrease body mass index [11, 12]. While these investigations demonstrate the utility of pedometers to promote physical activity in the general population, their value in preventing weight gain in college students is still relatively unknown.

The impact of steps on body weight during the freshman year was evaluated by LeCheminant et al. [13]. The results from this study demonstrated that there was no difference over the academic year in weight gain between students randomized to achieve 10,000 steps per day and those in the control group [13]. However, this study did not assess the steps of the students at baseline in the control or intervention groups and failed to evaluate the steps of the participants in the control group throughout the duration of the study. As a result, researchers speculated that it might be possible that students were already achieving 10,000 steps per day, so the intervention might not have been enough to prevent weight gain.

The purpose of this investigation was to evaluate the impact of 3 progressively higher step recommendations on preventing weight and body fat gain during the first university academic year (24 weeks) in women. The step recommendations were 10,000, 12,500, and 15,000 steps per day; 12,500 and 15,000 represent a 25% and 50% increase in daily physical activity. Additionally, this study sought to control other potential confounding factors of increased physical activity, such as eating behaviors, and diet. The original hypothesis of the study was that there would be a dose response relationship between step recommendation and body weight, with higher numbers of steps predicting less weight and fat weight gain over 24 weeks compared to lower numbers of steps.

## 2. Methods

### 2.1. Design

The study was a three-arm randomized trial. Participants were randomized to one of three step recommendations that included 10,000, 12,500, and 15,000 steps per day (6 days per week) over 24 weeks. These step recommendations represented an incremental 25% increase in physical activity. Participants were provided encouragement and support to achieve the recommended level of steps; however, no participant was dropped for not achieving the step recommendation. All participant identification numbers were randomized at the beginning of the study by the senior investigator using PC-SAS. The randomized intervention allocation list was kept concealed from study personnel by the senior investigator until baseline testing was complete and the participant number was assigned. Once the participant number was assigned, the intervention allocation was revealed by the senior investigator. The study was approved by the institution review board and all participants gave informed consent before participating.

### 2.2. Participants

Participants included 120 college women aged 18–22 years who were in their first year of college. A health history questionnaire was used to determine participants' health status and ability to participate in moderate-to-vigorous activity (MVPA) without limitations. Additionally, participants who were up to six months postpartum or who were planning to become pregnant in the next 8 months were excluded from the study. Women taking any medications that alter metabolism, who had a body mass index below 18.5 kg/m^2^, or who took more than 11,000 steps per day at baseline were excluded from the study. The 11,000-step exclusion was set to prevent participants from being randomized into the 10,000-step group who were already exceeding the step counts of the other groups [14]. We used 11,000 steps instead of 10,000 steps to exclude participants because we assumed that physical activity would be higher at the beginning of school, as students tend to have more free time and were getting used to campus life. Following randomization into one of the three step groups, participants were instructed not to discuss their step count assignment with other participants. The modes of recruitment included flyers, classroom announcements, booths, social media, and word of mouth.

### 2.3. Procedures

Once study eligibility was confirmed, baseline assessments were completed. The baseline assessment included three 24-hour dietary recalls, a DXA scan, anthropometric measurements of weight and height, and physical activity. To assess physical activity, participants were given an accelerometer and pedometer and instructed to wear them for 4 consecutive days. Accelerometers were used to supplement pedometers because accelerometers provide richer data describing physical activity patterns, such as time spent in sedentary, light, and MVPA.

Following the baseline assessments, if the person's average daily steps exceeded 11,000 per day, the participant was dropped from the study. Eligible participants were then randomized to a step count. Participants were instructed to meet their assigned step goal 6 out of 7 days of the week, and text messages were sent to the participants every day to encourage them to meet their step goal. They were not, however, restricted to their step count assignment since this seemed unreasonable. Participants returned to the lab every two weeks to download their step count information and to complete a sham questionnaire about stress. This questionnaire was used to divert attention away from body weight so that participants did not anticipate a weight-related outcome for the study.

In addition to health and stress questionnaires, participants also completed ten 24-hour multiple-pass dietary recalls throughout the duration of the study. Three recalls were performed randomly at baseline and an additional recall was completed every 4 weeks during the intervention period (7 times total). The day of the week for this recall was chosen randomly.

All the assessments that were performed at baseline were completed again either at the end of the intervention or during the intervention period. Physical activity was assessed continually using an assigned pedometer, and for 4 consecutive days during weeks 20 to 22, using an accelerometer. Weight, height, and body composition were assessed during the last week of the intervention.

### 2.4. Measurement of Anthropometrics

Height was measured to the nearest 0.1 cm using a wall-mounted stadiometer (SECA, Chino, CA). Weight was measured to the nearest 0.1 kg using a digital scale (Tanita, Arlington Heights, Illinois). GE iDXA (GE, Fairfield, CT) was used to assess fat-free mass, fat mass, lean mass, percent body fat, and visceral adipose tissue [15–17]. Visceral fat was calculated using the CoreScan application of the GE iDXA [18, 19]. Calibration of the DXA scan took place at the beginning of each testing day using a manufacturer-provided calibration block. Scans were analyzed using Encore software version 17.

### 2.5. Measurement of Physical Activity

Participants were issued an Omeron HJ-720-IT pedometer. Pedometers have been used in research to both encourage and track steps [10]. They were instructed to wear the pedometers throughout the day for four days at baseline (two weekdays and two weekend days) and for the duration of the 24 weeks of the study [20]. The pedometer was worn at all times during the study, with the exception of showering or other water related activities. This data was downloaded every two weeks.

ActiGraph GT3X accelerometers were also used to assess physical activity and were worn by the participants over a four-day period (two weekdays and two weekend days) at baseline and then again between weeks 20 and 22 of the intervention period. The participants were instructed to wear the accelerometers on their hip at the level of the umbilicus and above the anterior superior iliac spine, opposite the hip of the pedometer. They were also instructed to wear the accelerometer day and night with the exception of water activities. Accelerometers give information on time and intensity of activity and have been shown to be accurate in determining differences in low-moderate and high-intensity activity levels [21–24]. The day's data was considered complete if they wore the accelerometer 75% of the time between 7 a.m. and 11 p.m. Nonwear time was conservatively defined as any string of at least 20 consecutive minutes with zero acceleration. Data was collected in 60 second epochs. Physical activity intensity levels were categorized using the following cut-points: vigorous activity (>5999 counts/min), moderate activity (2020–5999 counts/min), light activity (100–2019 counts/min), and sedentary (0–100 counts/min) [25].

### 2.6. Measurement of Diet

Dietary intake was assessed by multiple uses of the Automated Self-Administered 24-hour Dietary Recall (ASA24) survey provided by the U.S. National Cancer Institute in conjunction with the National Institutes of Health (NCI and NIH). Using the Web-based ASA24, the participants were asked to record everything they ate and drank in the previous 24 hours. The ASA24 includes a database of food items separated by food group. The participants were instructed by both audibly and by typed prompts in how to record meals and snacks. The serving size choices were supplemented with pictures. The system ensured that participants had included all foods consumed during the previous 24 hours by using repeated prompts for “oft-forgotten foods,” such as ketchup, soda, and butter. ASA24 has little error variance compared to interviewer-based recalls and is more cost-effective [26, 27].

### 2.7. Data Analysis

Sample size was estimated a priori and based on the comparison between the 10,000- and 15,000-step groups for body weight. We estimated a 2 kg weight difference between groups with a common standard deviation of 2.8 kg [28]. Beta was set at 0.20 and alpha was set at 0.05. Based on these assumptions, 33 participants were needed in each group to have 80% power. We anticipated a 15% dropout rate and thus recruited 40 participants in each group for a total of 120 participants.

Means and standard deviations were reported for all variables of interest. A mixed effects repeated measures analysis of variance was used to analyze the results of physical activity, body composition, and diet. Results were evaluated to assess the interactive and main effects of period and step group (10,000, 12,500, and 15,000 steps). A similar analysis was performed to evaluate pedometer measured average steps per day by step group and month of the study. The least squared means procedure was used to further evaluate any significant main and interactive effects. For both, the accelerometer-measured physical activity and diet, assessment days were averaged across the period (baseline and follow-up) prior to analysis. PC-SAS version 9.4 was used for the mixed effects modeling.

## 3. Results

### 3.1. Participants

**Figure 1:**
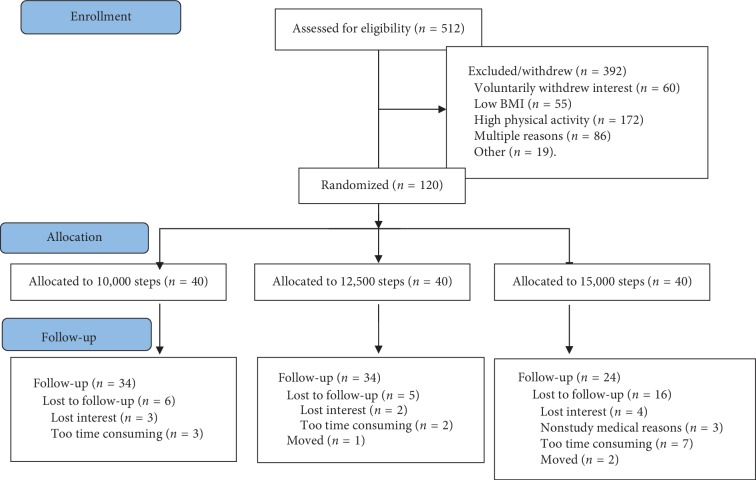
Participant flow diagram.

Characteristics of the participants are described in Figure 1 and Table 1. A total of 512 women were assessed for eligibility and 120 were randomized into one of the three step groups (10,000 steps (*n* = 40), 12,500 steps (*n* = 40), and 15,000 steps (*n* = 40)). Of the 120 participants, 92 completed the study. Dropout was not equal between the groups with the 15,000-step group having the highest dropout rate. However, there were no differences in age, BMI, percent body fat, steps per day, or energy intake between participants who finished the study and those who did not.

### 3.2. Steps/Physical Activity

**Figure 2:**
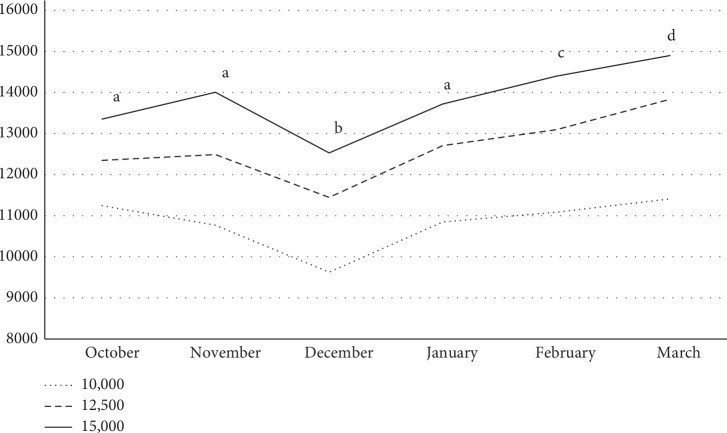
Average steps per day by month over the course of 24 weeks in 1st year college women. There was no month by step recommendation interaction observed. The average steps per day for all three step recommendation groups were different (*P* < 0.05). a, b, c, d: average steps per day in months with different letters are significantly different (*P* < 0.05). For example, average steps in October were higher than those in December but lower than those in February and March.

Figure 2 displays the pedometer downloaded steps per day over the course of the study. The average daily steps accumulated by each step count were 10,786 ± 1501, 12,650 ± 2001, and 13,762 ± 2098 steps per day for the 10,000-, 12,500-, and 15,000-step groups, respectively, and all three counts were significantly different from the others (*P* < 0.0001). There was no significant step-count-by-month interaction observed. However, average steps per day tended to increase over the course of the study with the exception of December, which resulted in the lowest averages of the study for all three groups (see Figure 2).

Accelerometer physical activity data are presented in Table 2. A period (baseline or follow-up) by step group interaction was observed for nonsedentary time, MVPA, and steps but was not significant for light PA (*P*=0.0505). Follow-up tests revealed that nonsedentary time (all light activity and MVPA) did not change from baseline to follow-up in the 10,000-step group but increased by 43 minutes per day in the 12,500-step group and by 77 minutes per day in the 15,000-step group. MVPA changed significantly from baseline in all three groups, increasing by 15 minutes, 28 minutes, and 34 minutes in the 10,000-, 12,500-, and 15,000-step groups, respectively.

### 3.3. Body Composition and Weight

Table 3 shows data collected from the DXA scans in relation to body composition and weight. On average women gained 1.5 ± 2.3 kg over the course of the study (*F* = 37.61, *P* < 0.0001) with no significant difference between the 10,000-, 12,500-, and 15,000-step groups. DXA scans showed that 0.7 ± 1.7 kg of this weight gain was fat and 0.8 ± 1.1 kg was lean body mass (Ps < 0.001). Thus, weight gain was 44% fat mass and 56% lean. Visceral adipose tissue mass and volume were low and did not change over the course of the 6 months. There was no period-by-step-group interaction for any body composition variable.

### 3.4. Diet

Table 4 describes the dietary data for participants who completed the study. The average total caloric intake at baseline was 2115 ± 527 kcal. There was no main effect for period or step group and there were no interactive effects between period and step group. There was no main effect in total calories consumed from baseline to follow-up. The total fat and CHO intakes were not statistically different between groups. However, the difference in protein intake was significant (*P* < 0.05), with the 12,500-step group consuming less protein at follow-up than the other groups.

## 4. Discussion

The goal of the study was to evaluate if progressively exceeding the recommended step count of 10,000 steps per day (in 25% increments) would attenuate weight and fat accumulation in college freshmen women. Regardless of the overall increase in steps per day and consistent separation in steps between the three step groups, women gained about 1.5 kg as a whole over the 6 months of the study (Table 3). The average weight gain observed was within the range of weight gain previously reported in other studies of college students. While the “Freshman 15” has been proven to be a myth for most people, a 1 to 4 kg average weight gain is commonly observed during the first academic year [1, 2, 8, 29]. These results also do not seem to be explained by diet, since there was no difference in energy intake between the three step groups. However, like most assessments of dietary intake, underreporting is common and interindividual variability tends to be large which may account for the lack of any observed difference between the three conditions.

The lack of attenuation in weight gain between step groups was surprising, since physical activity progressively increased with each step recommendation and physical activity increases energy expenditure and alters energy balance. One limitation in using steps to increase physical activity is that all steps were counted the same regardless of the intensity of the activity being performed. Thus, light steps were counted the same as vigorous steps. This could explain the lack of separation in weight gain between groups, because the majority of the increased activity in this study came from light activity and there was no change in vigorous activity. Thus, one explanation for the lack of separation in weight gain between groups might be that the activity was less intense and did not alter energy balance sufficiently to have a meaningful impact on body weight. Another explanation could be due to the normal physical maturation that occurs within this age group.

LeCheminant et al. evaluated how the recommendation of 10,000 daily steps impacted weight gain during the freshman year in women. Both the intervention and control groups gained a similar amount of weight (1.0 ± 2.5 kg) [13]. These results are similar to those observed in our study, but we observed no impact of steps beyond the 10,000-step count recommendation. It is possible that exceeding a certain step count does not result in more benefit on body weight or fat. An observation study of college women demonstrated that steps per day predicted body weight up to around 11,000 steps per day and that going beyond this level of habitual steps did not predict body weight or body fat [6]. Our study supports these findings and suggests that altering step recommendations may not be sufficient to prevent weight gain during the freshman year.

Step goals of 10,000 and 12,500 seemed to be well tolerated and achievable by most participants. However, exceeding 12,500 steps per day was more challenging to achieve. Even though achieving this recommendation was a struggle, the 15,000-step recommendation resulted in significantly more steps on average than was seen with lower recommendations.

While this study did observe weight gain, the composition of the weight gain was primarily lean tissue (56% lean tissue and 44% adipose tissue). This was unexpected, since a higher proportion of weight gain as lean mass is atypical and other studies tend to indicate that weight gain in college is primarily realized as body fat rather than lean mass. For example, Morrow et al. found that the composition of the weight gain observed during the freshman year in women was 73% fat [8]. It is possible that since 10,000 steps per day is considered “Active” [25], and all the women in the study were assigned at least 10,000 steps per day, there might be a benefit for all the women in the study. While the classification of “Active” makes no reference to the quality of steps being taken, women who get 10,000 steps per day in this study accumulated 67 minutes of MVPA daily on average.

Although weight was not affected by the intervention in the study, there was a positive impact on physical activity patterns that may have other emotional and health benefits. One positive outcome of the study was that sedentary time was drastically reduced in both the 12,500- and 15,000-step groups over the academic year. In the 15,000-step group, sedentary time decreased by as much as 77 minutes/day. While this may not have been sufficient to prevent weight gain, the benefits of decreasing sedentary time have been shown in other studies to reduce cardiometabolic and inflammatory biomarkers [30] as well as reduction in risk of anxiety and depression [31, 32].

While our study makes a significant contribution to the literature on the effects of physical activity on college weight gain, it is important to address the limitations that are associated with the study. One limitation of this study was that there was no control group. This limits the interpretation of the study, since we are unable to determine if the weight gain observed in the study would have been more or less without any intervention. Additionally, we did not evaluate step counts lower than 10,000 step per day. It is possible that lower step recommendations may have allowed us to see more effects on weight, or if there was a plateauing effect on weight after 10,000 steps per day. Another limitation was that there was an unequal dropout rate and attrition in the 15,000-step group was more drastic than the 10,000- and 12,500-step groups, even though no participant was dismissed from the study for not meeting their step goals. While this level of attrition should be considered when interpreting the results of the study, it is a common problem with demanding physical activity interventions [33]. In addition, adherence to the 15,000-step count was challenging, especially at the beginning of the study. Participants were more compliant to their step recommendations over time but as a group never averaged 15,000 steps per day. Also, participants were not restricted on the number of daily steps. Participants were given a minimum goal, but they could exceed this goal. There were participants in the 10,000- and 12,500-step counts who consistently exceeded their step recommendation. However, it was not feasible to tell the participants to stop walking after they met their assigned step goals. Finally, measuring diet is difficult and underreporting energy intake is common. While 24-hour recalls are nonreactive and have been validated for assessing dietary intake, all methods of measuring diet have their limitations.

Our study makes a meaningful contribution to the literature on freshman weight gain by evaluating three different step recommendations of progressively higher volumes and these recommendations progressively increased by 25%. This is supported by objectively measured and downloaded step data for every day of the study rather than subjective self-reported data. In addition, our study was unique in that it looked at attenuating weight gain in a population at risk of weight gain rather than weight loss. Finally, we used accelerometers to better describe the quantity and quality of the step interventions.

## 5. Conclusions and Future Research

The results of this study suggest that going progressively beyond 10,000 steps per day has a positive impact on physical activity patterns but does not prevent weight gain in freshman women. Setting step goals can habitually increase MVPA and decrease sedentary time. In fact, the recommendation of 15,000 steps per day resulted in over an hour more activity each day. Although prevention of weight gain was not achieved, the reduced sedentary time was substantial and has benefits that likely extended beyond weight management.

Preventing weight gain during the freshman year continues to be an important topic of research. Weight gain is accelerated during this transitional time in life. Results from this study suggest that increasing habitual steps per day is not sufficient to accomplish this goal. Future research will likely have to evaluate different dietary and behavioral strategies together to prevent weight gain or evaluate physical activity of a greater volume and/or intensity.

## Figures and Tables

**Table 1:** Demographic data by step group at baseline.

	10,000 steps (*n* = 40)		12,500 steps (*n* = 40)		15,000 steps (*n* = 40)		Combined (*n* = 120)		*F*	*P*
	*M*	SD	*M*	SD	*M*	SD	*M*	SD		
Age (yrs)	18.0	0.2	18.1	0.3	18.0	0.4	18.1	0.3	0.36	0.69
BMI (kg/m^2^)	23.5	2.7	22.7	2.14	23.2	2.6	23.1	2.5	0.87	0.42
Body fat %	34.0	5.3	32.7	4.1	33.0	5.2	33.2	4.9	0.79	0.45
Average steps at baseline	8082	1608	8375	1537	8213	1444	8225	1512	0.35	0.71

*Note. F* and *P* values refer to the comparison of groups at baseline.

**Table 2:** Physical activity data by step group measured by actigraphy.

	10,000-step group (*n* = 34)				12,500-step group (*n* = 34)				15,000-step group (*n* = 24)				*F*	*P*
	Baseline		Follow-up		Baseline		Follow-up		Baseline		Follow-up			
	*M*	SD	*M*	SD	*M*	SD	*M*	SD	*M*	SD	*M*	SD		
Steps per day	9232	2141	11066^*∗a*^	2360	9992	907	13638^*∗b*^	2135	9571	2264	14557^*∗c*^	2338	9.52	<0.001
Aerobic steps	3887	1764	5351^*∗a*^	2043	4638	1876	7601^*∗b*^	2373	4603	1597	7619^*∗b*^	2028	4.94	0.009
Nonsedentary (min)	309.1	61.3	322.2^*∗a*^	59.3	313.9	64.2	353.7^*∗b*^	57.5	301.6	43.7	370.7^*∗b*^	57.6	6.59	0.002
Light activity (min)	255.6	56.0	254.8	58.5	257.3	66.6	269.8	62.8	246.4	45.3	282.5^*∗*^	58.5	3.10	0.050
MVPA (min)	53.5	56.0	67.5^*∗a*^	17.5	56.7	17.5	84.2^*∗b*^	19.1	55.3	21.4	88.7^*∗b*^	17.7	6.63	0.002

*Notes. F* and *P* values refer to the period-by-step-group interaction. ^*∗*^Significant difference from baseline to follow-up (*P* ≤ 0.05). ^a,b,c^Means with different letters were statistically different at follow-up (*P* < 0.05). There was no mean difference in any of the variables at baseline.

**Table 3:** Body composition results by step group.

	10,000-step group (*n* = 34)				12,500-step group (*n* = 34)				15,000-step group (*n* = 24)				*F*	*P*
	Baseline		Follow-up		Baseline		Follow-up		Baseline		Follow-up			
	*M*	SD	*M*	SD	*M*	SD	*M*	SD	*M*	SD	*M*	SD		
BMI (kg/m^2^)	23.5	2.7	23.9^*∗*^	2.2	22.7	2.1	23.2	2.2	23.2	2.6	23.7	2.0	0.15	0.857
Body fat %	34.1	5.3	34.0	4.5	32.7	4.4	33.1	4.1	32.9	5.2	33.5	3.9	0.92	0.401
Total mass (kg)	65.0	7.6	66.3^*∗*^	5.8	61.5	6.8	63.0^*∗*^	6.8	63.6	8.2	65.3^*∗*^	7.1	0.27	0.762
Total fat mass (kg)	22.4	5.8	22.8	4.7	20.2	4.1	21.0^*∗*^	3.9	21.2	5.3	22.0^*∗*^	4.2	0.66	0.519
Total lean mass (kg)	40.2	3.1	41.0^*∗*^	2.8	38.8	4.1	39.7^*∗*^	4.2	40.0	4.3	40.8^*∗*^	4.1	0.07	0.933
VAT mass (g)	153.0	131.0	154.1	149.2	118.3	94.6	128.0	128.4	154.0	112.2	167.8	108.7	0.46	0.634
VAT volume (cm^3^)	162.2	148.4	163.3	158.1	125.4	100.2	135.7	136.1	163.2	119.0	177.8	115.2	0.46	0.634

*Notes. F* and *P* values refer to the period-by-step-group interaction. ^*∗*^There was a significant main effect for period. Baseline means are different from follow-up (*P* < 0.05). There were no baseline differences between groups. There were no follow-up differences between groups.

**Table 4:** Baseline and follow-up diet by step group.

	10,000-step group				12,500-step group				15,000-step group				*F*	*P*
	(*n* = 34)				(*n* = 34)				(*n* = 24)					
	Baseline		Follow-up		Baseline		Follow-up		Baseline		Follow-up			
	*M*	SD	*M*	SD	*M*	SD	*M*	SD	*M*	SD	*M*	SD		
Kcals	2028	451	2056	396	2221	551	1954	347	2196	772	2139	381	1.81	0.17
Protein	72	19	76	24	79	22	65^*∗*+^	13	69	20	77	23	3.65	0.03
Carbohydrate	263	73	262	56	299	92	261	53	275	69	274	52	1.63	0.19
Total fat	79	22	81	17	82	26	75	16	95	75	85	21	0.26	0.77

*Note. F* and *P* values refer to the period-by-step-group interaction. ^*∗*^Statistically different from baseline in the 12,500-step group. ^+^Statistically different at follow-up in the 10,000-, 12,500-, and 15,000-step groups.

## Data Availability

The data used to support the findings of this study are available from the corresponding author upon request.

## Additional Points

*Implications and Contributions.* Weight gain is common during the first academic year of college. Physical activity has been found to have a positive impact on weight gain. Findings suggest that progressing beyond 10,000 steps per day has positive impacts on physical activity patterns but does not prevent weight gain in freshmen women.
